# Editorial: Towards an understanding of the cognitive mechanisms involved in threat processing and perception

**DOI:** 10.3389/fpsyg.2024.1427224

**Published:** 2024-05-29

**Authors:** Andras N. Zsido, Michael C. Hout, David S. March, Carlos M. Coelho, Jakub Polák

**Affiliations:** ^1^Psychology Institute, University of Pécs, Pécs, Hungary; ^2^Department of Psychology, New Mexico State University, Las Cruces, NM, United States; ^3^Department of Psychology, Florida State University, Tallahassee, FL, United States; ^4^Department of Psychology, University of the Azores, Ponta Delgada, Portugal; ^5^Department of Zoology, Charles University, Prague, Czechia; ^6^Department of Psychology, AMBIS University, Prague, Czechia

**Keywords:** attention, memory, fear, anxiety, disgust, perception, threat

## Part I - General introduction and the importance of the Research Topic

Much remains unknown about the cognitive mechanisms and information-processing biases involved in threat detection, and the acquisition and maintenance of threat associations. To complicate matters, these mechanisms and biases are likely to vary for different types of threats (see, e.g., Coelho et al., 2023), such as those posed by animals, weapons, social situations, or groups. There has been a recent push to identify ways to improve the methods used in research in this area, which has also led to reevaluation of theoretical frameworks (March et al., 2022; Landová et al., 2023; Zsido et al., 2024). It is therefore important to continue to elucidate the cognitive mechanisms (e.g., perception, attention, memory, learning) underlying threat processing in order to develop a better understanding of how they affect individual and social outcomes (Gober et al., 2021).

Research on the cognitive mechanisms involved in threat processing and perception can shed light on how our brains respond to threatening stimuli and provide insights into the fundamental processes underlying fear and anxiety (c.f., Clauss et al., 2022). This knowledge is essential for the development of effective interventions for anxiety disorders and phobias. A better understanding of these mechanisms may also help identify individuals who are more susceptible to exaggerated threat responses, allowing for targeted early interventions. Additionally, findings from this research can inform the design of environments and technologies that minimize unnecessary threat cues and promote psychological wellbeing.

Therefore, the goal of our Research Topic was to collect research articles that present empirical data and describe novel theoretical perspectives that address the effects of threats on cognitive processes. We sought to include research on how threat processing uniquely affects perception, attention, memory, attitude and evaluation formation, fear (un)conditioning, decision-making, planning and execution of defensive behaviors, and social processes. Another goal was to elucidate the social/cognitive processes that may play an important role in the etiology and maintenance of specific fears and phobias. Our Research Topic has collected nine papers that explore or elucidate the processes and mechanisms affected by threatening stimuli, with the overall goal of contributing to the field's understanding of the emergence, maintenance, modification, and expression of threat associations.

## Part II - Brief summary of the papers included in the topic

The first three studies (Apostolakis et al.; Ben-Baruch et al.; Xiao et al.) deepen our understanding of anxiety, fear perception, and the underlying cognitive processes involved therein. They highlight the importance of considering individual differences, cognitive strategies, and contextual factors in designing interventions and assessment tools for anxiety-related disorders.

Xiao et al. investigated the effects of reward-associative learning and traditional threat-avoidance training on anxiety and attentional bias. Their study focused on high trait anxious individuals and involved reward training or reward control training followed by Attention Bias Modification (ABM) training or control training. The results revealed that reward training reduced general anxiety and attentional bias. Interestingly, traditional ABM training only reduced anxiety when combined with reward training, suggesting a potential synergy between reward-based learning and traditional anxiety reduction techniques.

In Apostolakis et al.'s study, the researchers examined the psychometric properties of the abbreviated Social Phobia and Anxiety Inventory (SPAI-23) in Greek-Cypriot adolescents. They aimed to elucidate the dimensions of social fears in this population. Through exploratory factor analysis, they identified three social phobia factors and one agoraphobia factor, providing more nuanced insights into the assessment of social fears in adolescents. The findings contribute to refining assessment tools and understanding the multidimensional nature of social anxiety.

Ben-Baruch et al. explored the link between implicit and explicit emotion regulation and size estimation among women with arachnophobia. Their study delved into how emotion regulation strategies, such as reappraisal and suppression, influence perceptual biases in individuals afraid of spiders. While implicit emotion regulation did not directly impact size and valence ratings, the researchers found that greater use of reappraisal was associated with reduced negative feelings, whereas suppression was linked to increased size estimation of spider stimuli. These results shed light on the role of emotion regulation in modulating perceptual biases and offer potential avenues for the development of targeted treatments for specific phobias.

The subsequent four papers (Abado et al.; Kang and Osinsky; Peléšková et al.; Stolero et al.) collectively provide valuable insights into various aspects of human perception, attention, and emotional responses to threats, contributing to our understanding of human psychology in different contexts of danger.

Stolero et al. investigated differences and similarities in the perception of various risks (including extreme weather events, pandemics, and social disruption) between first responders and the public in several European countries. First responders tend to perceive higher risks for weather and natural events, while the public is more concerned about critical infrastructure dependencies and pandemics. The extent of these differences varies between countries, with Norway showing significant differences for all risks except extreme weather, while Sweden shows less variation. Understanding these differences is crucial to developing effective protective measures.

Kang and Osinsky studied attentional biases toward threatening faces in the context of social anxiety and explored methods to manipulate these biases. Using reward-based contingencies and neurophysiological measures, the researchers aimed to improve the efficacy and reliability of attentional bias modification (ABM) training. They found a general bias toward angry faces but observed variability in lateralization effects.

Abado et al. investigated the influence of a priori expectancies on the allocation of attention to phylogenetic (spiders) vs. ontogenetic (guns) threatening stimuli. Using a visual search array paradigm, the researchers manipulated expectancies and examined attentional biases toward these stimuli. Results indicate that while attentional bias was observed for spiders, it did not extend to ontogenetic threats such as guns. The results also replicated previous findings on attentional bias to spiders and revealed correlations between fear levels and attentional processes. The study highlights the role of expectancies and individual differences in shaping attention to different types of threat.

Similarly, Peléšková et al. investigated the evolutionary concepts of fear, disgust and anger responses to ancient and modern types of threat. The results suggest that modern threats elicit the strongest fear responses, while ancestral threats elicit the highest levels of disgust. Interestingly, modern threats such as toxic substances mainly evoke fear and anger rather than disgust. Pandemic threats evoke both fear and disgust responses. The study suggests that ancient threats are not necessarily more powerful stimuli than modern threats, but they are highly specific, with snakes and heights being particularly prominent fear factors.

Turning to snakes in the last two papers, Štolhoferová et al. investigated the fear response to snakes in individuals from Somaliland and the Czech Republic. They conducted experiments using a picture-sorting approach with 48 snake species, including venomous viperids and elapids. The results showed significant agreement between the Somali and Czech respondents, with vipers eliciting the highest levels of fear in both populations. Interestingly, fear scores for vipers were consistently higher than for deadly venomous elapids, and snake body width emerged as a significant predictor of fear. This suggests that evolutionary, cultural and cognitive factors contribute to the fear response to snakes.

Frynta et al. studied the effect of snake threat displays on spontaneous human attention. They conducted an eye-tracking experiment on populations in Somaliland and the Czech Republic to determine whether human attention is drawn to snakes in threatening postures. The results showed that participants in both regions showed increased attention to snakes in threatening postures compared to relaxed postures. The study also found a significant effect of snake morphotype, with cobras eliciting the most attention, followed by vipers, while other morphotypes showed less significant effects. Despite cultural and environmental differences, the overall pattern of responses to snakes was similar in both populations, supporting the evolutionary origin of the phenomenon.

## Conclusion

Collectively, this set of articles represents an important step forward in our understanding of the cognitive processes underlying threat processing and perception. Much work is left to be done because this topic touches such a broad swath of cognition, from the everyday processing of threats that are encountered, to the clinical and social impacts of disorders including anxiety and phobias. Our hope is that this Research Topic will answer some unresolved questions, will stimulate new questions and theoretical outlooks, and will raise awareness of the need for more research in these areas.

## Author contributions

AZ: Writing – original draft, Writing – review & editing. MH: Writing – original draft, Writing – review & editing. DM: Writing – original draft, Writing – review & editing. CC: Writing – original draft, Writing – review & editing. JP: Writing – original draft, Writing – review & editing.
